# FT4-to-FT3 ratio is a novel prognostic marker in subacute combined spinal cord degeneration patients

**DOI:** 10.1515/tnsci-2022-0340

**Published:** 2024-05-03

**Authors:** Song Luo, Xiao-rui Wang, Li-juan Yang, Liang-yu Zou

**Affiliations:** Department of Neurology, The First Affiliated Hospital of Bengbu Medical University, Bengbu 233004, China; Department of Pediatrics, The First Affiliated Hospital of Bengbu Medical University, Bengbu 233004, China; Department of Neurology, Shenzhen People’s Hospital, The Second Clinical Medical University, Jinan University, Shenzhen 518020, China

**Keywords:** subacute combined spinal cord degeneration, thyroid hormone, FT4/FT3 ratio, clinical outcome, prognostic marker

## Abstract

**Objectives:**

The FT4-to-FT3 ratio (FFR) variations in patients with subacute combined spinal cord degeneration (SCSD) as a potentially useful prognostic indicator are still unknown. This study aimed to investigate the changes of FFR as a potentially valuable prognostic predictor in patients with SCSD.

**Methods:**

This study included 144 consecutive SCSD patients who received standard diagnostic and therapeutic procedures between January 2015 and December 2021 and were admitted to the Department of Neurology at the First Affiliated Hospital of Bengbu Medical University. At the time of admission, we gathered data on all patients’ demographics, daily routines, previous chronic conditions, medication histories, and other clinical details. For the purpose of measuring FFR, blood samples were specifically taken within 48 h of admission. The degree of neurological impairment of patients was assessed using the functional disability scale at the time of admission. At 6 months following discharge, the Modified Rankin Scale (mRS) was used to evaluate the clinical prognosis. To evaluate the relationship between the FFR and the risks of a poor outcome (mRS > 2), univariate and multivariate logistic regression analysis was utilized. The significance of the FT4/FT3 ratio in predicting the clinical outcomes in SCSD patients 6 months after discharge was assessed using the area under curve-receiver operating characteristic (AUC-ROC).

**Results:**

About 90 patients (62.5%) of the 144 patients had poor outcomes, while 54 (37.5%) had favorable outcomes. Higher FFR at admission was independently linked to higher odds of a poor outcome, according to a logistic analysis. With an optimized cutoff value of >2.843, the FFR exhibited the maximum accuracy for predicting a poor outcome, according to the AUC‒ROC curve (AUC 0.731, *P* < 0.001; sensitivity, 77.8%; specificity, 83.3%). FFR was identified as an independent predictor of poor outcomes by multivariate logistic regression (OR, 2.244; 95% CI, 1.74–2.90; *P* < 0.001).

**Conclusions:**

We discovered that in patients who had a bad result 6 months after discharge, the FFR had dramatically increased at the time of admission, providing a unique prognostic marker in patients with SCSD.

## Abbreviations

SCSD: subacute combined spinal cord degeneration
TH: thyroid hormone
FFR: FT4/FT3 ratio
FDS: functional disability scale
FT3: free tri-iodothyronine
FT4: free thyroxine
TSH: thyroid stimulating hormone
EMG: electromyogram
mRS: modified Rankin Scale

## 1 Background

Subacute combined spinal cord degeneration (SCSD) is a rare demyelinating disease caused by metabolic disorders [1,2]. Some of the recognized causes include inadequate vitamin B12 (Vit-B12) intake and malabsorption [2], deficiency of or abnormality in the cobalt transporter in the blood [3], immunological factors [3], and deficiency of folic acid and nicotinic acid [2,4]. Hypothyroidism, which results in demyelination and axonal degeneration of nerve fibers [2,5,6,7,8,9], inhibits the methylation reaction *in vivo*. Thyroid hormones have a favorable effect on peripheral nerve regeneration and are involved in the maturation, development, and normal function of the central nervous system [5]. Since a higher free thyroxine (FT4)/free tri-iodothyronine (FT3) ratio specifically reflects the decline in tissue-specific deiodinase activity and has been demonstrated to be a novel prognostic marker in several neurodegenerative diseases [10,11], we hypothesized that it would likely be relevant in SCSD patients.

## 2 Objectives

To investigate the evolution of the FT4-to-FT3 ratio (FFR) in patients with SCSD as a potentially valuable prognostic predictor, we retrospectively collected and analyzed clinical data from patients with SCSD. The relationship between thyroid hormone levels and the clinical prognosis of these patients was evaluated using laboratory indices, imaging data, and functional assessment scores. Potential influencing factors were also identified.

## 3 Methods

### 3.1 Object

**Figure 1:**
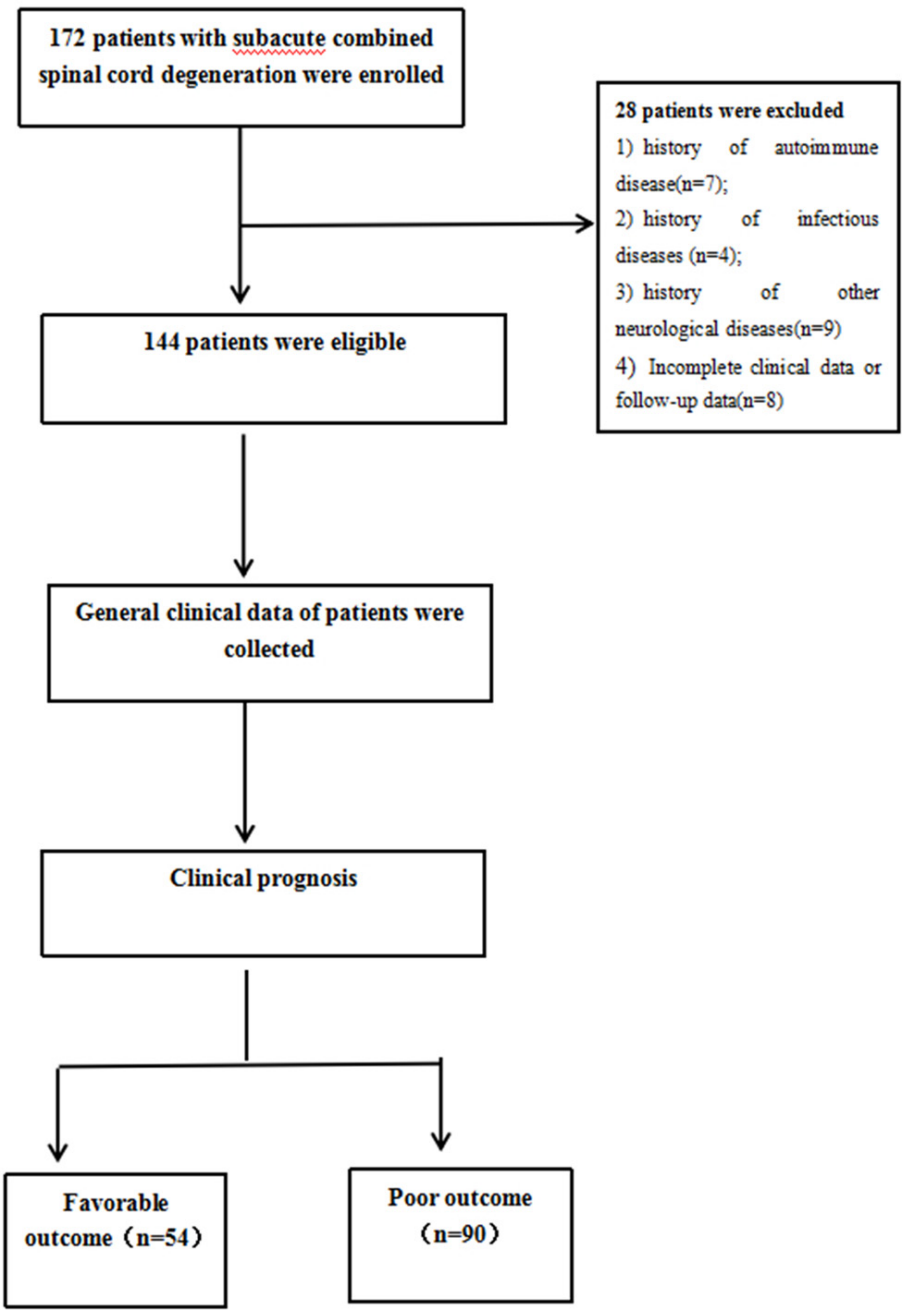
The flow chart of the study. A total of 172 patients were diagnosed with SSCD in our hospital between January 2015 and December 2021. Of these, 28 patients were excluded: seven had a history of autoimmune disease, four had a long history of infection, nine had a history of other neurological diseases, and eight had incomplete clinical data or had been lost to follow-up. Thus, a total of 144 patients were enrolled, and pertinent clinical data were gathered. 3 months later, patients were classified into two groups: those with favorable prognosis (*n* = 54) and those with a bad prognosis (*n* = 90).

About 144 patients with SCSD between January 2015 and December 2021 were recruited at the Department of Neurology at the First Affiliated Hospital of Bengbu Medical University. We performed this retrospective observational cohort study. Inclusion criteria were a) patients who first came to our hospital and met the revised SCSD diagnostic criteria [12]; b) chronic or subacute disease onset with slow progression; c) damage to the lateral cord and posterior cord or peripheral nerve; d) evidence of serum Vit-B12 deficiency, or elevated/normal levels of Vit-B12 with symptom relief after Vit-B12 supplementation; and e) absence of any other spinal cord or peripheral nerve disease. Exclusion criteria included a) history of uremia, heart failure, or autoimmune disease; b) history of acute/chronic inflammatory demyelination of multiple nerves, history of myelitis or spinal cord trauma, history of exposure to toxins or poisoning, or history of chemoradiotherapy for malignancies; c) history of infectious diseases including viral hepatitis, tuberculosis, syphilis, or AIDS; d) history of stroke, encephalitis, or other neurological diseases. The flow chart for the study is shown in Figure 1.

### 3.2 Clinical data

**Figure 2:**
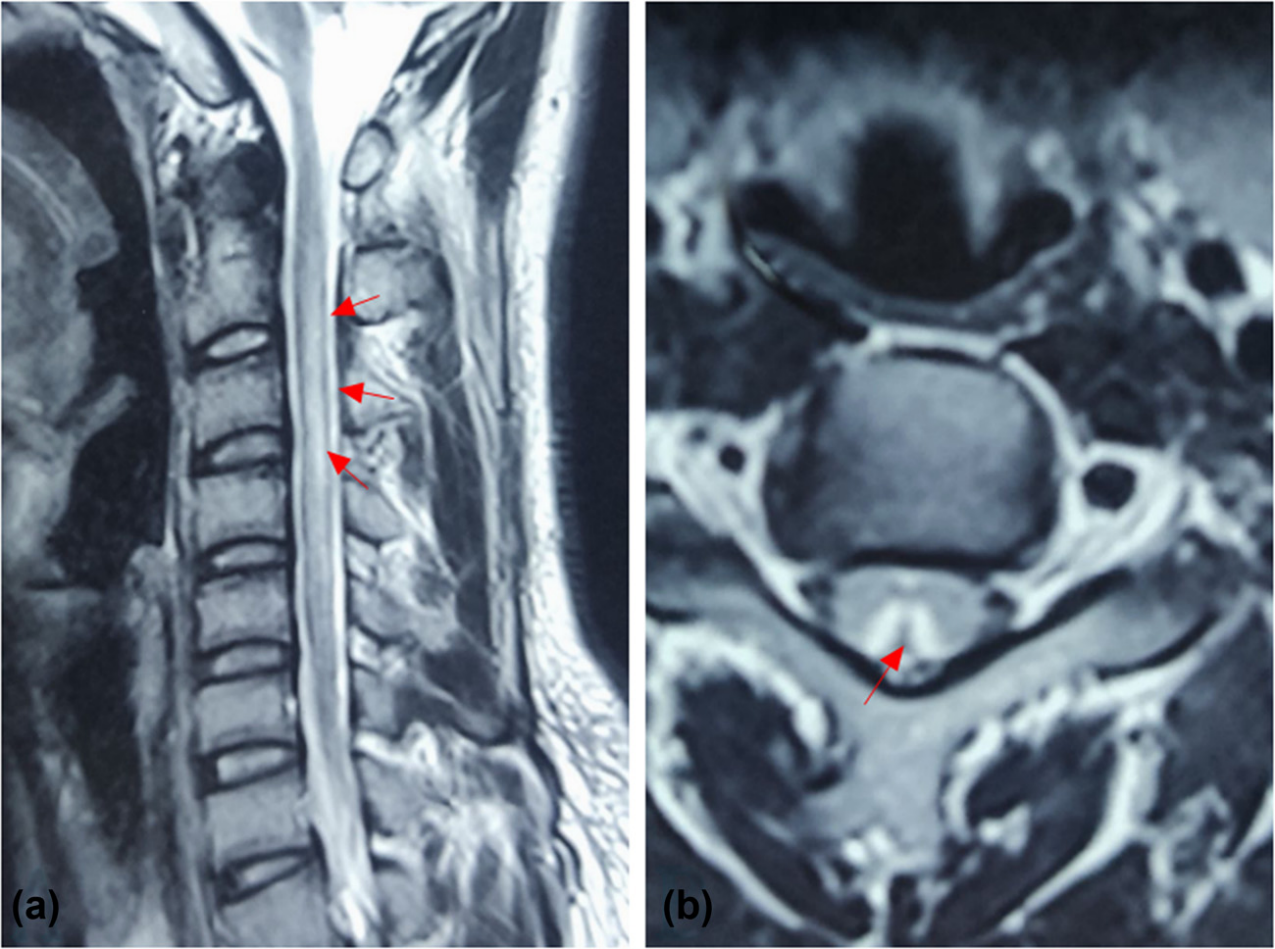
Cervical MRI T2WI images of a 19-year-old male patient showing abnormal signals of the cervical spinal cord in sagittal view (indicated by red arrow, a), and typical “inverted V sign” of the cervical spinal cord in transverse view (indicated by red arrow, b).

Age, sex, smoking and alcohol consumption history, and history of digestive disorders were obtained from patients. Regular blood tests, biochemical analyses, thyroid function tests (FT3, FT4, thyroid stimulating hormone (TSH)), FFR, serum levels of Vit-B12, folic acid, lipids, and uric acid, as well as electromyograms (EMG) and medulla spinalis magnetic resonance imaging (MRI) data were meticulously recorded. Sagittal and axial T1-weighted and T2-weighted (T2WI) MRI images of the spinal cord were obtained (phase-arrayed coil, superconducting MRI system, 3.0T, Siemens, Germany). Each MRI scan was read by two radiologists, and a senior neurologist examined each case (Figure 2). In case of any disagreement among the doctors, a re-assessment was conducted. The thyroid function profile was measured using a direct chemiluminescence method (ADVIA Centaur XP, Siemens, USA). The normal reference ranges for TSH, FT3, and FT4 levels are 0.37–4.94 mIU/L, 3.10–6.80 pmol/L, and 12.0–22.0 pmol/L, respectively. To determine hypothyroidism, TSH > 4.94 mIU/L and/or FT3 3.10 pmol/L and FT4 12.0 pmol/L were employed [10]. The normal ranges for folic acid and Vit-B12 are 3–20 ng/mL and 180–914 pg/mL, respectively (determined by chemiluminescence immunoassay).

### 3.3 Quantitative scores of clinical outcomes

The functional disability scale (FDS) [12] was used to evaluate the level of neurological impairment at the time of admission. The following were the score items: a) Gait: normal, 0 score; difficulty standing with eyes closed, 1 score; abnormal gait without assistance, 2 score; walking with assistance, 3 score; patient confined to a wheelchair or bedridden, 4 score. b) Sensation: no sensory disorder, 0 score; sensory impairment in toes and fingers, 1 score; sensory disorders below the ankle and wrist, 2 score; sensory disturbance in the upper and lower extremities, 3 score. c) Cognitive function: normal, 0 score; living without social assistance despite intellectual or mental disability, 1 score; partial dependence in daily life, 2 score; and total dependence for daily living, 3 score. d) Nerve reflex: normal, 0 score; decreased or absent ankle reflex, 1 score; decreased or absent knee reflex, 2 score; and decreased or absent upper limb tendon reflex, 3 score. e) Pyramidal bundle function: normal, 0 score; Babinski sign positive, 1 score; spastic paraplegia of lower limbs, 2 score; and spastic paraplegia of the extremities, 3 score. The score ranged from 0 to 16, with a higher score reflecting more severe nerve injury.

**Figure 3:**
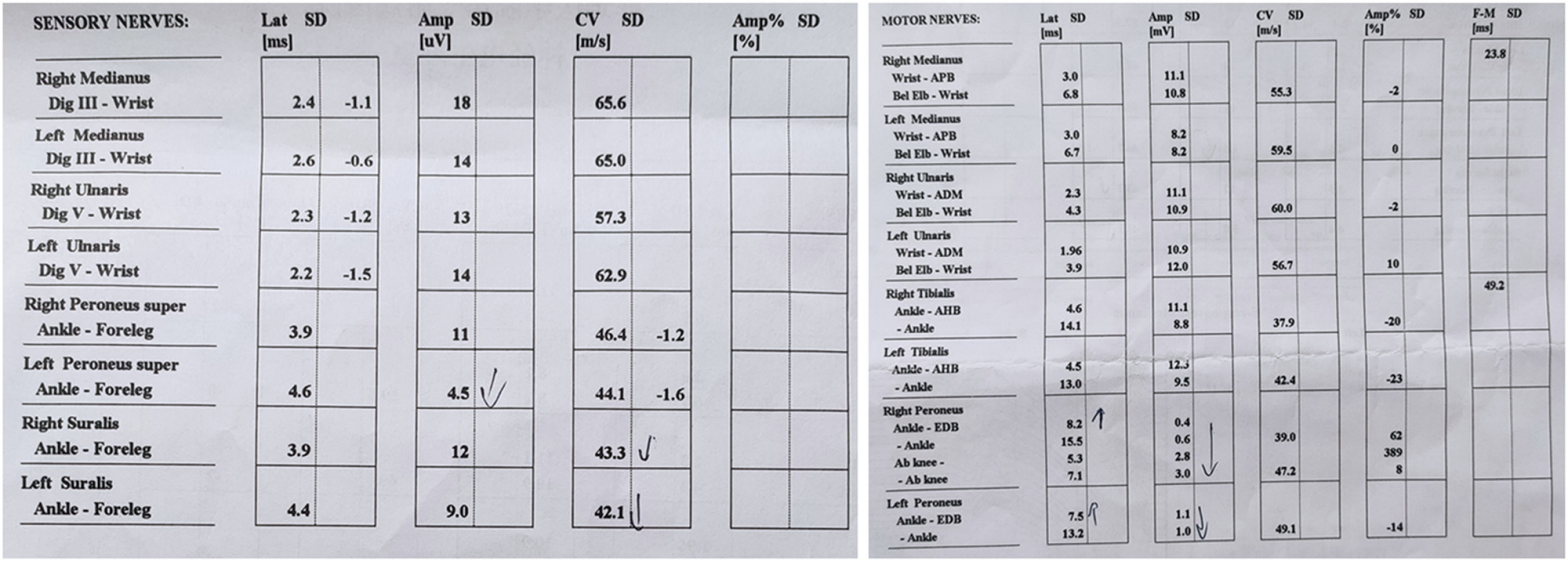
EMG of a 25-year-old woman showing peripheral nerve damage in both lower limbs.


Based on the modified Rankin Scale (mRS) score [13] (0–2: favorable outcome; 3–5 or death: poor outcome), the link between hormone levels and patients’ clinical outcomes was established. About 6 months following discharge, patients with SCSD were contacted telephonically to obtain relevant information. This prospective observational study was approved by the Ethics Committee of the First Affiliated Hospital of Bengbu Medical College. A formal statement of informed consent was signed by each participant or their authorized representative (Figure 3).

### 3.4 Statistical analysis

The statistical analysis was performed using IBM SPSS Statistics 25 Software. Continuous variables with normal distribution were presented as mean ± standard deviation, while non-normally distributed continuous variables were presented as median and interquartile range (25–75th percentile). Categorical variables were shown as frequency (%). To evaluate between-group differences, the *t*-test, Chi-squared test, and Man Whitney U rank sum test were applied, as needed. A logistic regression model was used to assess how baseline characteristics affected clinical prognosis. Variables with *P*-values <0.05 in the univariate analysis were included in the multivariable logistic regression analysis, and the forward stepwise regression approach (a predictor selection algorithm) was used to compute the odds ratio value and 95% confidence interval of the related factors. The multicollinearity was examined by variable inflation factors. The assumption of linearity of independent variables and log-odds was evaluated by the Box‒Tidwell test. The area under the receiver operating characteristics curve (AUC‒ROC) was used to evaluate the significance of the FFR in predicting clinical outcomes in SCSD patients. Hosmer‒Lemeshow goodness-of-fit test (HL test) and Nagelkerke’s *R* ^2^ were used to assess the model goodness of fit. Statistics were considered significant for *P*-value <0.05.

**Ethical approval:** The research related to human use has been complied with all the relevant national regulations, institutional policies and in accordance with the tenets of the Helsinki Declaration, and has been approved by the author’s institutional review board or equivalent committee. This study was approved by the Ethics Committee of the First Affiliated Hospital of Bengbu Medical College.
**Informed consent:** Informed consent has been obtained from all individuals included in this study.

## 4 Results

### 4.1 General clinical characteristics

**Figure 4:**
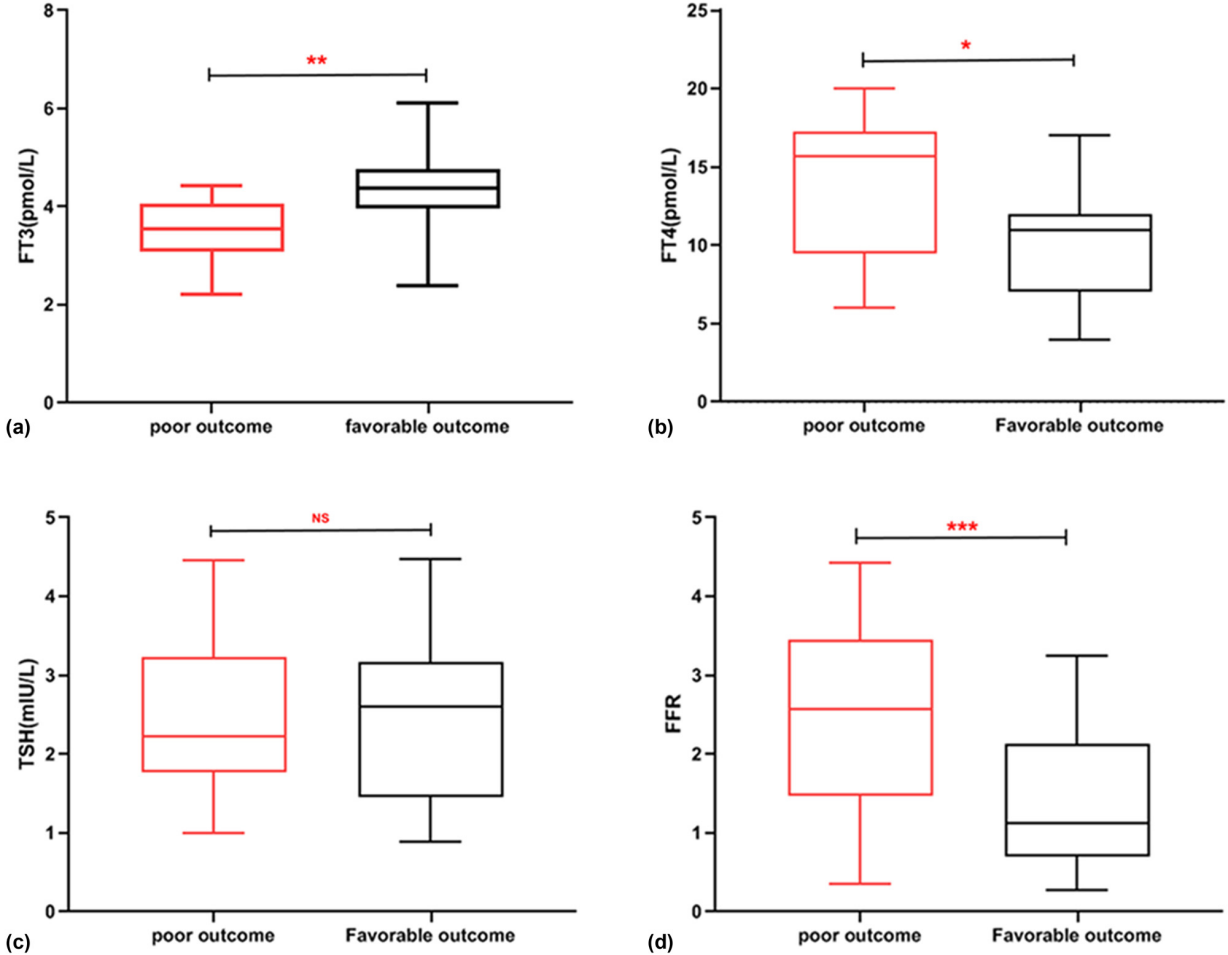
Boxplots showing (a) FT3 levels, (b) FT4 levels, (c) TSH levels, and (d) FFR levels of the poor outcome and favorable outcome groups of SSCD patients. Red represents the poor outcome group and black represents the favorable outcome group. The central line indicates the median value. Max and min values are represented by the whiskers, and the top and bottom box lines indicate Q1 and Q3. (ns, no significant; *P < 0.05, **P < 0.01, ***P < 0.001; *t* test).

Initially, 172 SCSD patients were enrolled; of these, 28 patients were subsequently dropped due to exclusion criteria, leaving just 144 patients. Among them, 54 patients (37.5%) had a favorable outcome, while 90 patients (62.5%) had a poor prognosis. The EMG was found to be more sensitive than the medulla spinalis MRI, which is in line with previous studies [14], as abnormal signals were found in 32 patients (59.3%) by EMG and 14 patients (25.9%) by MRI in the group with a favorable outcome. Poorer outcomes were associated with alcohol intake, gastrointestinal disorders, low Vit-B12 and folic acid levels, hypothyroidism, and higher FDA scores (all *P* < 0.05, Table 1, *t*-test). Regarding thyroid hormone levels, the group with poorer outcomes had significantly higher FT4 (14.05 ± 1.12 vs 10.24 ± 1.01, *P* = 0.001, *t*-test), FT3 (3.48 ± 0.69 vs 4.34 ± 0.85, *P* = 0.002, *t*-test), and FFR (2.63 ± 0.15 vs 1.42 ± 0.28, *P* = 0.001, Table 1, *t*-test). However, TSH did not reach statistical significance between the two groups (2.39 ± 0.14 vs 2.50 ± 0.31, *P* = 0.732, Figure 4, *t*-test).

**Table 1:** Clinical characteristics of the patients enrolled in the study

Factors	Group			Normality test	Variance homogeneity test	*t* Value	*t-*Test
	Total	Poor outcome	Favorable outcome				
		(mRS > 2)	(mRS ≤ 2)	*P*-value	*P*-value		*P*-value
*N* (%)	144	90 (62.50)	54 (37.50)	0.236	0.261	0.142	0.887
Age (years), Median (IQR)	62.92 ± 16.12	64.28 ± 17.09	63.87 ± 16.22				
**Sex**							
Female, *n* (%)	78 (54.2)	48 (53.3)	30 (55.6)	0.543	0.147	2.103	0.147
**History of smoking,** ***n*** **(%)**							
No smoking	86 (59.7)	52 (57.3)	34 (63.0)	0.269	0.349	0.193	0.661
History of alcohol consumption, *n* (%)							
No	84 (58.3)	48 (53.3)	36 (66.7)	0.612	0.79	4.662	0.032*
Hypertension, *n* (%)	48 (33.3)	34 (37.8)	14 (25.9)	0.562	0.395	1.633	0.201
Diabetes, *n* (%)	39 (27.1)	29 (32.2)	10 (18.5)	0.152	0.632	2.553	0.114
Gastrointestinal disease, *n* (%) no	101 (70.14)	57 (63.3)	44 (81.5)	0.342	0.291	4.476	0.031*
Vitamin B12, *n* (%) reduced	90 (62.5)	62 (68.9)	28 (51.9)	0.645	0.437	4.18	0.041*
Folic acid, *n* (%) reduced	64 (44.4)	48 (53.3)	16 (29.6)	0.219	0.812	6.75	0.012*
Hypothyroidism, *n* (%)	30 (20.8)	24 (26.7%)	6 (11.1%)	0.165	0.541	4.053	0.041*
Triglyceride (mmol/L)	1.11 ± 0.56	1.31 ± 0.57	1.17 ± 0.47	0.89	0.409	1.521	0.132
Cholesterol (mmol/L)	3.59 ± 1.18	3.79 ± 0.82	4.01 ± 0.59	0.521	0.317	1.721	0.087
HDL (mmol/L)	1.03 ± 0.34	1.06 ± 0.29	1.00 ± 0.40	0.679	0.24	1.04	0.301
LDL (mmol/L)	2.15 ± 0.79	2.03 ± 0.63	2.27 ± 0.93	0.93	0.412	1.844	0.073
Uric acid (µmol/L)	277.08 ± 94.28	280.89 ± 83.43	263.28 ± 72	0.467	0.325	1.275	0.203
MCV (fL)	96.30 ± 10.93	97.04 ± 10.69	96.57 ± 11.47	0.241	0.165	0.249	0.803
HB (g/L)	121 ± 22.47	120.11 ± 21.24	121.89 ± 24.23	0.231	0.121	0.462	0.652
FT3 (pmol/L)	3.91 ± 0.88	3.48 ± 0.69	4.34 ± 0.85	0.567	0.372	6.629	0.002*
FT4 (pmol/L)	13.15 ± 1.39	14.05 ± 1.12	10.24 ± 1.01	0.643	0.785	7.451	0.001*
TSH (mIU/L)	2.44 ± 0.23	2.39 ± 0.14	2.50 ± 0.31	0.434	0.56	1.213	0.732
FT4/FT3 ratio	2.20 ± 0.35	2.63 ± 0.15	1.42 ± 0.28	0.271	0.311	7.97	0.001*
FDS	6.83 ± 2.41	8.33 ± 1.41	5.33 ± 2.38	0.419	0.879	7.778	0.001*
Medulla spinalis MRI, *n* (%)	52 (36.1)	38 (42.2)	14 (25.9)	0.471	0.677	2.284	0.131
EMG, *n* (%)	109 (75.7)	77 (85.6)	32 (59.3)	0.783	0.409	12.685	0.001*

*P < 0.05.

### 4.2 Association between the FT4/FT3 and Clinical prognosis

**Figure 5:**
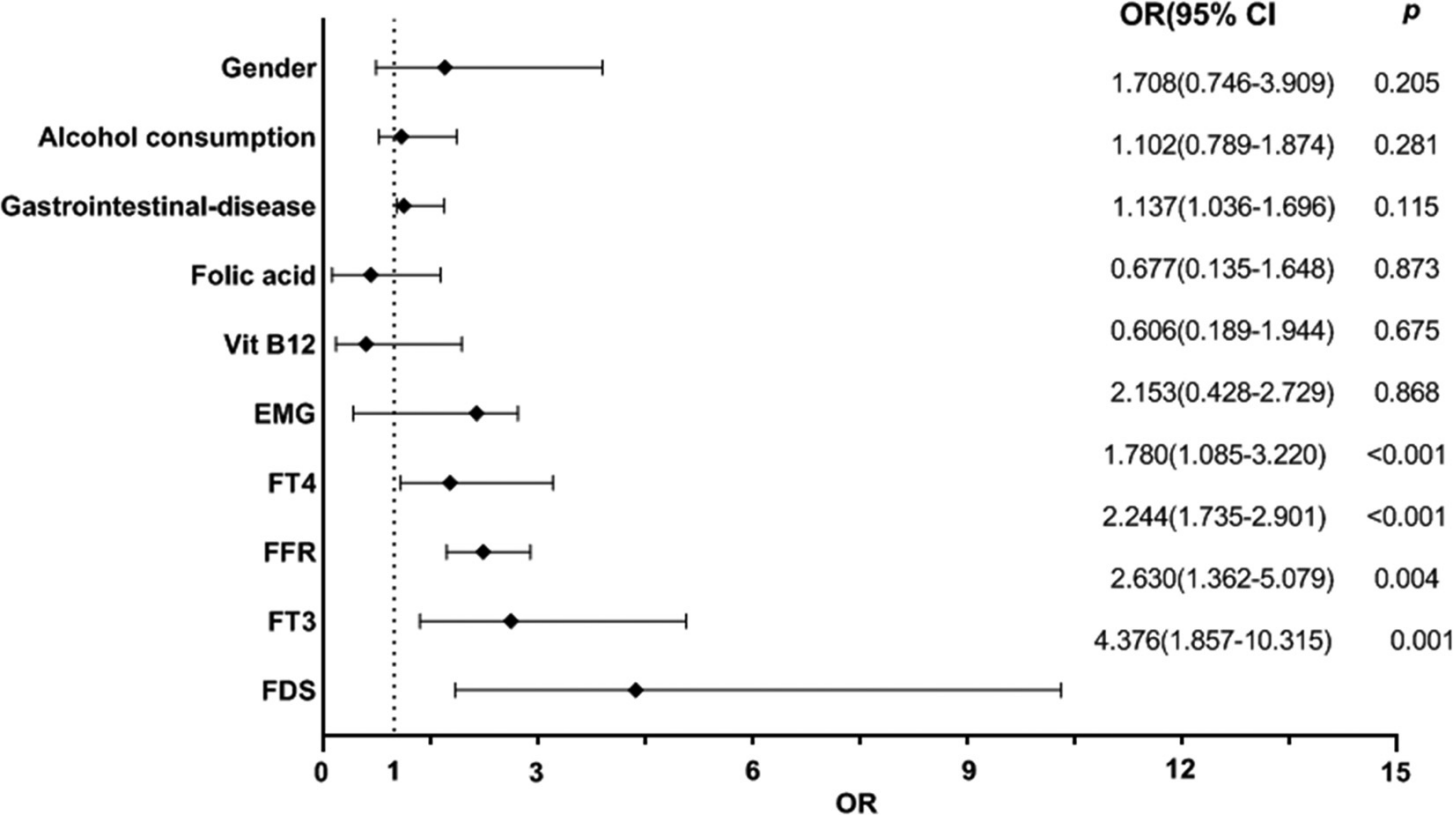
Predictors of poor outcomes in SSCD patients from multivariable binary logistic regression analysis based on FFR, FT3, FT4, FDS, gender, alcohol consumption, gastrointestinal disease, Vit-B12, Folic acid, as well as EMG. CI, confidence interval; OR, odds ratio.

Binary logistic regression analysis was applied to explore potential clinical prognostic factors in patients with SCSD. Multivariate analysis revealed that higher FFR (OR = 2.244; 95% CI: 1.735–2.901; *P* < 0.001) and FDS (OR = 4.376; 95% CI: 1.857–10.315; *P* = 0.001) were independently associated with an elevated risk of poor outcome (Figure 5). The HL test was utilized for validation, with *P* = 0.046 for FFR and *P* = 0.429 for FDS. Moreover, FFR and FDS had Nagelkerke’s *R* ^2^ of 0.572 and 0.821, respectively. However, gender, alcohol usage, gastrointestinal disease, Vit-B12, folic acid, and EMG were not found to be statistically significant, according to the results of the multivariate logistic regression analysis (Figure 5).

### 4.3 FFR combined with FDS score to predict clinical prognosis

**Figure 6:**
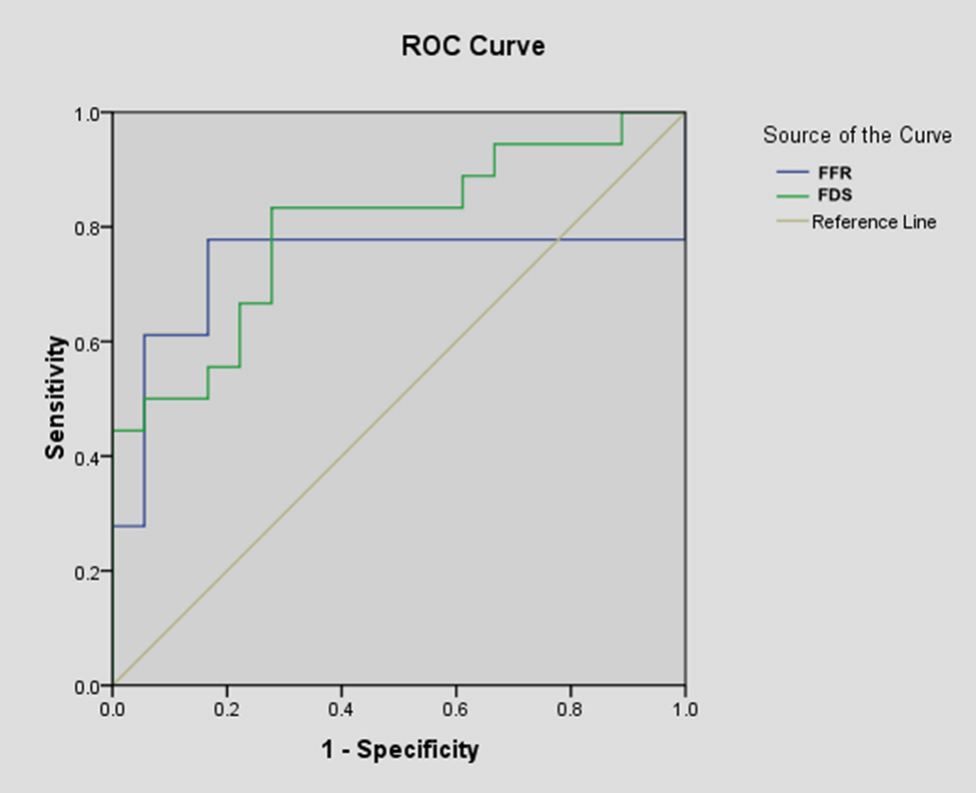
ROC Curves of FDS score and FFR in predicting poor clinical outcome. Cutoff value and corresponding sensitivity and specificity are shown. ROC, receiver operating characteristic; AUC, area under the curve.

The FFR and FDS scores were used as detection variables, and the poor outcome (mRS > 2) served as the outcome variable. The relative AUC values of FFR and FDS scores were 0.731 (95% CI: 0.636–0.827) and 0.796 (95% CI: 0.723–0.869), respectively (Figure 6). The optimal value of FFR for predicting clinical prognosis was 2.843, with a sensitivity of 77.8% and specificity of 83.3% (Figure 6). These data indicated that the aforementioned indications can accurately predict the clinical prognosis 6 months after discharge.

## 5 Discussion

The pathological hallmarks of SCSD are demyelination and degeneration of nerve fibers, which can damage peripheral nerves and brain white matter, and are more prevalent in the posterior column and lateral column of the spinal cord [15]. The neuroendocrine hormone, thyroid hormone, is essential for several metabolic processes, including the production of energy, cell survival and proliferation, and nervous system maintenance [16].

Previous studies have shown that hypothyroidism can lead to Vit-B12 deficiency, which eventually results in SCSD. The development, maturation, and maintenance of central nervous system as well as the regeneration of the peripheral nervous system are all aided by thyroid hormones [17]. Thyroid hormone analogues have been shown in recent studies [18,19] to promote myelin regeneration in the brain and spinal cord [14] as specific central nervous system precursor drugs. Additionally, the development and maintenance of the digestive system are impacted by thyroid hormone levels [15]. Thyroid hormone supplementation can help alleviate the neurological symptoms of the disorder [16].

Variations in thyroid hormone levels may be a rare and curable cause of SCSD, according to research on the potential diagnostic and prognostic value of FFR. The main objectives of this study were to investigate the FFR and its relationship with outcomes in SCSD patients. Comparative analysis of the data from the poor and good outcome groups revealed that the prevalence of alcohol consumption, Vit-B12 deficiency, gastrointestinal disease, and folic acid deficiency were higher in the poor outcome group. This is in line with the findings of Talhada et al. and suggests that an unhealthy lifestyle will eventually have a negative impact on the clinical prognosis of SCSD patients [19].

Furthermore, our preliminary analysis of medulla spinalis MRI and EMG in SCSD patients revealed that the group with poor outcomes had a higher percentage of aberrant spinal MRI and EMG than the group with favorable outcomes. It was also assumed that the EMG may be more sensitive to detecting neurological impairment in SCSD patients, which is consistent with the findings of Akiyuki Hiraga and other researchers [20]. When compared to individuals who had favorable outcomes, we found that patients with poor outcomes had lower FT3 and higher FFR and FT4. A previous study revealed that decreased FT3 and elevated FT4 levels were independent predictors of long-term mortality risk of hospitalized patients with chronic Non-thyroidal illness syndrome [21]. Our result showed that the FFR was an independent risk factor for poor prognosis. However, due to the study’s small sample size, results for other conventional risk factors for the nervous system, including smoking, hypertension, and hyperlipidemia, were all comparable. It has not been investigated whether the FFR can predict long-term clinical outcomes for SCSD patients. Our study collected FT4 and FT3 level data after undergoing standardized therapy and found an independent relationship between the FFR and poor outcomes.

Last but not least, it is currently recognized that FDS can be used to assess the neurological deficit status of SCSD patients in order to determine the clinical prognosis [22]. Given that there is some subjectivity, it is reasonable to find more objective predictors. We found that FFR was just as helpful as FDS for assessing the clinical outcomes of SCSD patients when we used the AUC‒ROC curve to examine the FFR and FDS scores. The AUC‒ROC curve results in some accuracy predicting clinical prognosis. However, more patients should be included in the future study. Taniguchi et al. identified a link between lower FFR levels and a better clinical outcome (mRS 2) in their study of SCSD patients receiving therapy [23]. Therefore, it is crucial to understand the role of FFR during SCSD and identify the window of time during which the FFR is reliable as a predictor of prognosis [24]. Patients with poor outcomes, according to our study, had FFR levels of more than 2.843. The following factors may contribute to this mechanism. Thyroid hormones regulate the development, maturation, and normal function of the central nervous system as well as peripheral nerve regeneration. Hypothyroidism, in particular FT3, which can cause demyelination and axonal degeneration of nerve fibers [25–27], can impair the methylation process *in vivo*. Lower FT3 levels over the course of the SCSD period can exacerbate neuronal loss and the accompanying neurological deficits [28]. In addition, Hashimoto’s thyroiditis, one of the most common autoimmune thyroid diseases, may cause spinal cord degeneration when complicated with encephalopathy [29].

Some limitations still exist in this current study. First, since this study was a single-center retrospective analysis, selection bias might have been present. Larger multicenter investigations are required for more conclusive evidence. Second, subsequent research should include some novel biological nerve functioning markers linked to thyroid function, such as neurotrophic factors, to increase the validity of our findings. In addition, the common autoimmune thyroiditis auto-antibodies, including anti-thyroglobulin (TG) and anti-thyroid peroxidase (TPO) antibodies, were not involved in the subsequent analysis due to their inaccessibility. However, considering that an autoimmune background of possible thyroid hormone fluctuations cannot be ruled out, further research about the role of anti-thyroid antibodies in SCSD will be conducted. Finally, the mechanisms underlying this still need to be completely understood and further explored.

In conclusion, the degree of neurological impairment in patients with SSCD may be predicted by hypothyroidism to some extent. SCSD patients with concurrent hypothyroidism and increased FFR must receive treatment as soon as possible to prevent neurological deterioration and improve prognosis. The FFR significantly enhanced at admission in patients with SSCD who had a poor outcome 6 months after discharge. This study provides a novel prognostic marker. However, it still needs more clinical investigation in the future.
